# In Vitro Reconstitution of a Cellular Phase-Transition Process that Involves the mRNA Decapping Machinery**

**DOI:** 10.1002/anie.201402885

**Published:** 2014-05-26

**Authors:** Simon A Fromm, Julia Kamenz, Erik R Nöldeke, Ancilla Neu, Georg Zocher, Remco Sprangers

**Affiliations:** Max Planck Institute for Developmental BiologySpemannstrasse 35, 72076 Tübingen (Germany); Friedrich Miescher Laboratory of the Max Planck SocietyTübingen (Germany); Interfaculty Institute of Biochemistry, University of TübingenTübingen (Germany)

**Keywords:** NMR spectroscopy, phase transitions, processing bodies, protein–protein interactions, self-assembly

## Abstract

In eukaryotic cells, components of the 5′ to 3′ mRNA degradation machinery can undergo a rapid phase transition. The resulting cytoplasmic foci are referred to as processing bodies (P-bodies). The molecular details of the self-aggregation process are, however, largely undetermined. Herein, we use a bottom-up approach that combines NMR spectroscopy, isothermal titration calorimetry, X-ray crystallography, and fluorescence microscopy to probe if mRNA degradation factors can undergo phase transitions in vitro. We show that the Schizosaccharomyces pombe Dcp2 mRNA decapping enzyme, its prime activator Dcp1, and the scaffolding proteins Edc3 and Pdc1 are sufficient to reconstitute a phase-separation process. Intermolecular interactions between the Edc3 LSm domain and at least 10 helical leucine-rich motifs in Dcp2 and Pdc1 build the core of the interaction network. We show that blocking of these interactions interferes with the clustering behavior, both in vitro and in vivo.

Processing bodies (P-bodies) are cytoplasmic micrometer-scale ribonucleoprotein (RNP) foci that were initially identified in mammalian cells^1^ and that have now been observed in distant branches of eukaryotes. The list of proteins that are found in these mRNP granules has grown significantly^2^ and comprises general mRNA decay factors including the Dcp2 decapping enzyme and the enhancers of decapping Dcp1, Edc3, Pat1, and LSm1–7.1c, 3 The proposed roles of the clustering of the decapping enzyme within a confined cellular space includes enhanced substrate binding,^4^ buffering of the concentration of translating mRNAs,^5^ regulation of the concentrations of free cytoplasmic proteins,^6^ and a means to respond to cellular stress.^7^

The details of the interactions that underlie the self-assembly process of proteins and RNA into P-bodies are largely undetermined. Genetic studies have addressed the importance of individual proteins^8^ and revealed two major characteristics. 1) the details of the intermolecular interactions vary among species. For example, Edc3 and the Q/N rich C-terminal region of LSm4 can act as a scaffold for P-body assembly in *S. cerevisiae*,3c, 9 whereas the Pdc1 protein has been shown to be important in *S. pombe*.^10^ 2) the P-body assembly process is highly redundant and deletion of a single protein does not abolish the aggregation process completely.^8^ This redundancy complicates genetic approaches that aim to identify the interactions that regulate the self-assembly process.

Recently, it was shown that the purified Nck and N-WASP proteins can undergo a phase transition in vitro.^11^ These experiments are in line with the idea that the formation of cellular granules relies on a network of multivalent weak interactions between different components.^6^, ^12^ Herein, we ask whether it is possible to reconstitute a phase-transition process with proteins that have been shown to localize to P-bodies. To that end, we focus on the purified *S. pombe* Dcp2 mRNA decapping enzyme, its prime activator Dcp1 and the scaffolding proteins Edc3 and Pdc1 and show that these proteins can spontaneously self-assemble into oil-like droplets.

The proteins Edc3 and Dcp2 (Figure 1 A) have been shown to interact through contacts between the Edc3 LSm domain and short helical leucine-rich motifs (HLMs) in Dcp2.^13^ Herein, we use NMR spectroscopy titration experiments and show that Dcp2 contains at least seven different HLMs that are recognized specifically by the Edc3 LSm domain (Figure 1 B). Interestingly, the extent of the observed chemical shift changes varies, indicating that the HLM affinities vary considerably. To quantify these, we used isothermal titration calorimetric (ITC) assays (Figure 1 C,D) and find that the Edc3:HLM affinities range from (2.5±0.3) μm to the mm range.

**Figure 1:**
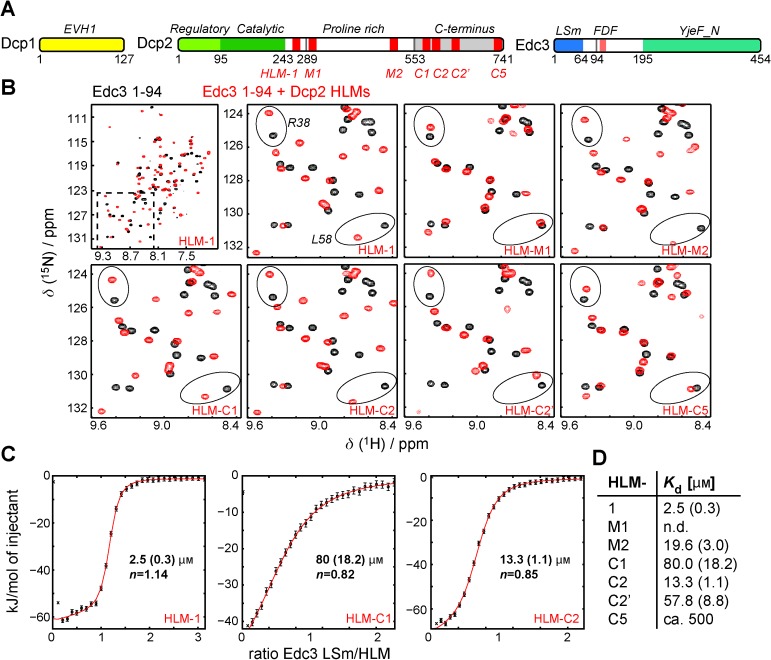
The seven HLMs in Dcp2 bind to the Edc3 LSm domain with a wide range of affinities. A) Schematic representation of *S. pombe* Dcp1 (EVH1 domain, yellow), Dcp2 (regulatory light green, catalytic dark green, HLMs red, proline-rich mid domain white, C-terminus gray) and Edc3 (LSm dark blue, FDF motif orange, YjeF N light blue). All used protein constructs are listed in Supporting Information Table S1 A. B) ^1^H–^15^N correlation spectra of the binding between the monomeric Edc3 LSm domain and the seven isolated Dcp2 HLMs. Black: free ^15^N labeled Edc3 LSm domain, Red: the Edc3 LSm domain in the presence of a fivefold excess of the individual Dcp2 HLMs. The boxed region in the top left panel is shown in all other spectra. Residues R38 and L58 are highlighted with ovals to indicate that the HLMs induce chemical shift perturbations to a different extent. C) ITC graphs for the binding of the Edc3 LSm domain to the Dcp2 HLM-1, HLM-C1 and HLM-C2 sequences. The best fit is drawn with a red line and the extracted *K*_d_ values including the error (standard deviation) are indicated. Deviations from *n*=1.0, where *n* refers to the stoichiometry, result from small inaccuracies in the protein-concentration determination. D) Overview of the determined *K*_d_ values (error in parenthesis) for the Dcp2 HLM:Edc3 interactions (n.d.: not determinable with ITC).

A prerequisite for protein phase separation is the capability to associate into indefinite soluble assemblies. In general, this condition is fulfilled for multivalent proteins with independent binding sites. Theoretically, Edc3 and Dcp2 can form such an indefinite and highly branched network of interactions because Edc3 is a dimer (and thus contains two LSm domains) and Dcp2 contains seven Edc3 docking sites (Figure S1 A in the Supporting Information). In addition, the binding affinities in the Edc3:Dcp2 system range from low μm to mm which has been suggested to be beneficial for the generation of phase transitions.^6^ To address if the Edc3:Dcp2 network is suitable to induce a phase separation in vitro, we mixed the recombinantly expressed and purified far C-terminal region of Dcp2 that contains four HLMs (Dcp2 residues 553–741; Table S1 A) with full-length Edc3. Interestingly, a clear phase separation can be observed using a bright-field microscope (Figure 2 A, left). Both Edc3 and Dcp2 are significantly enriched within the droplet-like structures as judged from the fluorescent signal that resulted from the Oregon green labeled proteins (Figure 2 A, and Figures S1 B, S1 C). Our data thus shows that the network of interactions between Edc3 and Dcp2 is sufficient to induce phase separations.

**Figure 2:**
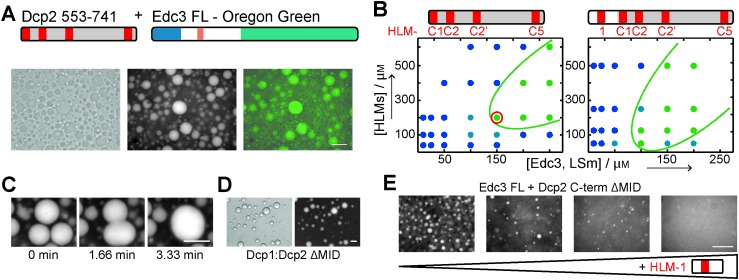
In vitro phase transitions of purified Edc3 and Dcp2. A) Phase transition of 50 μm Dcp2 553-741 and 150 μm Edc3 (doped 1:100 with Edc3-OregonGreen). Left: bright field (BF) channel; middle: Oregon Green (OG) channel; right: merge. B) Phase diagrams of phase transition of Edc3 together with Dcp2 553–741 (that contains 4 HLM sequences; left) or Dcp2 242–741ΔMID (that contains 5 HLM sequences). Given concentrations are modular concentrations, for example, 50 μm of Dcp2 553–741 is 200 μm HLMs, because 4 HLMs are in the Dcp2 553–741 construct. Modular LSm concentration is identical with total Edc3 concentration as an Edc3 monomer has one LSm domain. Occurrence of phase transition at a given condition is color coded; blue=no phase transition; light blue=beginning phase transition; green=clear phase transition. Green lines indicate progression of the phase boundaries. The red encircled condition is shown in (A) and (C). C) The in vitro droplets are highly dynamic and fuse over time. The time-scale is indicated below the OG channel pictures. D) Droplets formed by 25 μm Dcp1:Dcp2ΔMID and 100 μm Edc3 (doped 1:100 with Edc3-OG). Left: BF channel, Right: OG channel. E) Droplets formed by 25 μm Dcp2 242–741ΔMID and 100 μm Edc3 disappear by addition of increasing amounts of Dcp2 242–291 (containing HLM-1) (5 μm, 10 μm, 25 μm, 50 μm). Scale bars: 50 μm.

To obtain insights into the conditions that result in phase separation we varied the concentrations of Edc3 and the part of Dcp2 that contains 4 HLMs (Figure 2 B, left). We observed that phase separation occurs when a number of conditions are met. First, the absolute concentration of the individual proteins needs to be sufficiently high. In case a certain threshold concentration is not exceeded, phase separation does not take place and the proteins remain homogeneously distributed in solution. Second, the molar ratio of the two proteins needs to be within specific limits. In case the excess of either of the two proteins is too large, phase separation is abolished. This occurrence can be explained by the fact that a large excess of Edc3 would result in a situation where all HLMs are bound to a dimeric Edc3 protein, which would result in a loss of intertwining between different Dcp2 chains. A large excess of Dcp2 would, on the other hand, result in a situation where there are insufficient dimeric Edc3 proteins available to link the Dcp2 chains.

In a second set of experiments, we determined if the number of intermolecular interactions between components influences the phase-separation process. As full length Dcp2 that comprises seven HLMs was not expressed stably in our hands, we designed a version of Dcp2 that contains the complete unfolded C-terminal region but that lacks a part of the proline-rich region. This Dcp2 C-term ΔMid protein (Dcp2 residues 242–289+553–741; Supporting Information Table S1 A) contains five HLMs and undergoes phase transitions at concentrations that are significantly lower than those required for the Dcp2 construct that contains four HLMs. As an example: in the presence of 150 μm Edc3 phase separation takes place when the Dcp2 C-term ΔMid (five HLMs) concentration is 25 μm (125 μm modular HLM concentration), whereas double the concentration of the Dcp2-fragment that contains four HLMs (50 μm; 200 μm modular HLM concentration) is required. In agreement with previous observations,^11^ we can thus conclude that the valency and affinities of the interacting partners determines whether phase separation occurs at specific concentrations. Importantly, as controls we performed experiments using either a monomeric version of Edc3 or a Dcp2 sequence that only contains a single HLM (Figure S1 D,E). In none of these control experiments we observed phase separation (Figure S1 D,E), as the valency of one of the components is reduced to one.

Cellular foci, including P-bodies, have been shown to be highly dynamic.^14^ To determine if the in vitro Edc3:Dcp2 phase separations that we prepared from purified components display a similar behavior we monitored the droplets over a longer time. We observed a number of fusion events that confirm the liquid-like behavior of the in vitro droplets (Figure 2 C). This underscores that the in vitro system is similar to the in vivo situation regarding this aspect.

In vivo, the foci that contain Dcp2 and Edc3 also comprise numerous additional proteins. To test if it is possible to recruit additional proteins within the reconstituted in vitro droplets, we extended the Dcp2 C-term ΔMid construct such that it also includes the Dcp2 catalytic and the regulatory domain (Dcp2 ΔMid, residues 1–289+553–741; Table S1 A) that has been shown to interact with Dcp1.^15^ We then mixed purified Dcp1:Dcp2 complexes that harbored five HLM sequences with Edc3 and observed a clear phase separation, indicating that the Edc3:Dcp2 droplets can recruit additional factors (Figure 2 D). It should be noted that Dcp1 contains the binding site for Xrn1,^16^ thereby providing a mechanism to recruit the exonuclease to the Dcp1:Dcp2:Edc3 assemblies. In addition, the Edc3 protein contains an FDF repeat, providing a means to recruit the helicase DDX6/Dhh1.^17^ The catalytic domain of Dcp2^18^ and the helicase can then provide binding sites for mRNA substrates.

The Edc3:HLM interactions form the core of the in vitro droplets we assemble. Interference with this interaction is thus expected to result in the loss of phase separation. To test this hypothesis, we supplemented the Dcp2:Edc3 droplet conditions with the HLM-1 sequence (Figure 1 D). With increasing amounts of the HLM-1 Dcp2 peptide, we observe a disappearance of the phase separation in vitro (Figure 2 E). To assess the consequences of the specific interference with the Edc3:HLM interaction for P-body formation in vivo, we overexpressed the HLM-1 peptide in *S. pombe.* To observe the recruitment of Edc3 to P-bodies we replaced the *edc3+* gene at its endogenous locus by the *edc3+-mCherry* fusion construct. To simultaneously assess P-body integrity we additionally fused Dcp2 or Lsm7 to GFP at their endogenous loci. In cells that did not express the peptide that interferes with the Edc3:HLM interaction we observed P-bodies that contain Edc3-mCherry and Dcp2-GFP or Lsm7-GFP, respectively (Figure 3 A,B, top rows). On the other hand, in cells that overexpress the HLM peptide the Edc3-mCherry protein is no longer recruited into P-bodies (Figure 3 A,B, bottom rows). This lack of recruitment is most likely a consequence of the Edc3 LSm domains that are now saturated with the overexpressed mono-valent peptide. These in vivo observations are in agreement with our in vitro phase separation. Importantly, the overexpression of the HLM competition peptide does not result in the general loss of P-bodies as both Dcp2-GFP and Lsm7-GFP are still found in distinct cellular foci (Figure 3 A,B, middle panels, bottom rows). These observations stress the fact that in vivo P-body formation is a highly redundant process, where the loss of one P-body component does not result in a general loss of these foci.

**Figure 3:**
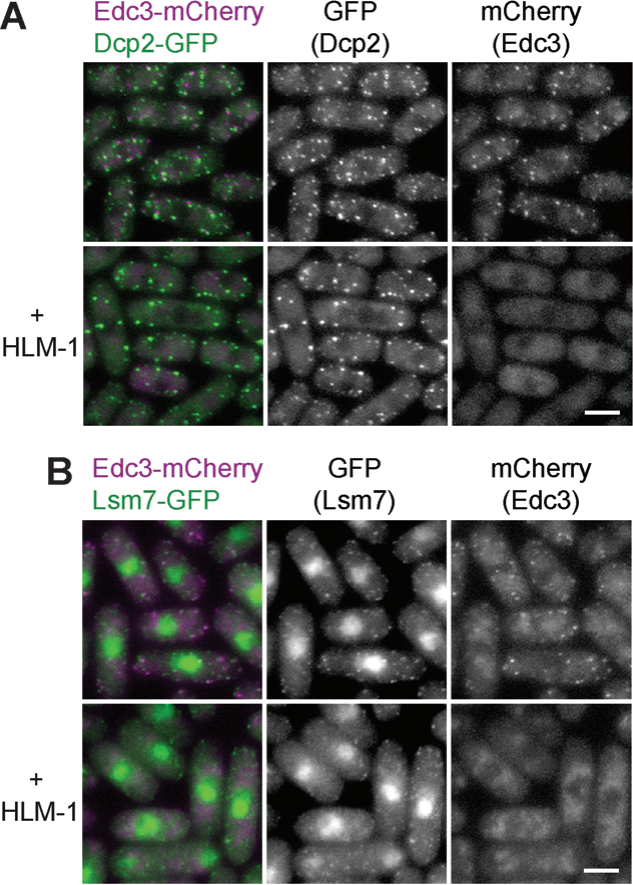
Effect of HLM-1 expression on Edc3 localization in vivo. A,B) Fluorescent micrographs of *S. pombe* expressing mCherry and GFP tagged versions of Edc3 and Dcp2 (A) or Lsm7 (B), respectively. Without HLM-1 overexpression Edc3 and Dcp2 are enriched into P-bodies (top row). Upon overexpression (*adh1* promoter) of HLM-1 Edc3 no longer localizes to P-bodies but is diffusely spread in the cell (bottom row). Dcp2-GFP (A) and Lsm7 (B) still localize to P-bodies upon overexpression of HLM-1, indicating that interference with the Edc3:HLM interactions does not generally disturb P-body formation. Scale bar: 5 μm.

To obtain insights into the redundancy of P-body formation, we turned to the Pdc1 protein that was recently implicated in P-body integrity in *S. pombe*.^10^ Pdc1 is related to metazoan Edc4 (Ge-1, Hedls)^19^ and both proteins contain an N-terminal WD-40 domain and a central coiled-coil region (Figure 4 A). Close inspection of the protein sequence at the N-terminal region of the *S. pombe* Pdc1 protein indicates the presence of several HLMs (Figure 4 A). Using NMR titration experiments (Figure 4 B) we confirmed that the N-terminal region of Pdc1 contains at least three HLM sequences that can interact with the Edc3 LSm domain. The extent of the induced chemical shift perturbations in the Edc3 LSm domain varied between the Pdc1 HLMs, as was the case for the seven Dcp2 HLMs (Figure 1 B, 4 B). Using ITC experiments, we quantified the associated affinities and found that the three Pdc1 HLM sequences interact with the Edc3 LSm domain between 150 μm and lower mM range (Figure 4 C,D). Interestingly, the Pdc1 protein contains a central coiled-coil region, through which the protein can oligomerize, resulting in an increased number of HLMs in the biological unit of the protein. Above, we have shown that a large number of HLMs is favorable for the phase separation process (Figure 2 B). To test whether Edc3 and Pdc1 are able to induce phase transitions in vitro we initially tried to purify the Pdc1 protein from *E. coli*. Unfortunately, full-length Pdc1 was, in our hands, not stable enough to allow for in vitro phase separation experiments. Consequently, we engineered a Pdc1 protein where the coiled-coil region was replaced with glutathione S-transferase (GST). GST is a dimer in solution and thus mimics the oligomerization effect of the Pdc1 coiled-coil region. We then used this designed dimeric Pdc1 protein (that has an HLM valency of six) and the dimeric Edc3 protein (that has an LSm valency of two) to test whether Pdc1 and Edc3 can undergo phase transitions in vitro. Interestingly, we observe that Pdc1 and Edc3 are able to engage in an indefinite network of intermolecular interaction that results in the formation of an oil-like droplet phase (Figure 4 E), as we observed for Dcp2 and Edc3 (Figure 2 A). We can thus conclude from our in vitro experiments that Pdc1 and Dcp2 are redundant proteins with regard to the potential of inducing phase separation when mixed together with Edc3.

**Figure 4:**
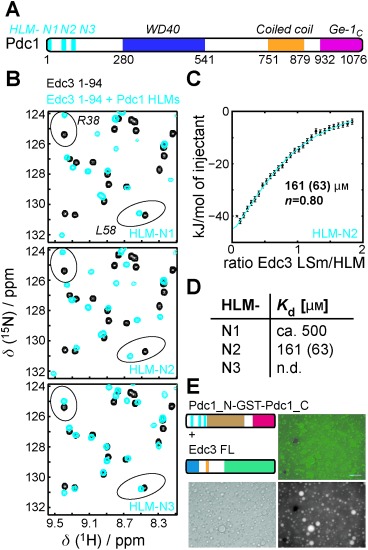
Pdc1 contains three HLMs at the N-terminus that bind to Edc3 with different affinities. A) Domain organization of the Pdc1 protein (N-terminal HLMs cyan, WD40 repeats purple, coiled-coil orange, Ge-1_C_-like domain (see below) pink). B) ^1^H–^15^N correlation spectra of the monomeric ^15^N labeled Edc3 LSm domain in the absence (black) and presence (cyan) of the individual Pdc1 HLMs. The boxed region in the top left panel of Figure 1 B is shown, ovals highlight two specific residues (see above). C) ITC graph of the Edc3 LSm domain binding to HLM-N2 from Pdc1. The best fit is drawn with a cyan line and the extracted *K*_d_ value is indicated. D) Overview of all *K*_d_ values (error in parentheses) determined for binding of Pdc1 HLMs to Edc3 LSm (n.d., not determinable with ITC). E) In vitro phase separation of designed dimeric Pdc1 construct that mimics full length Pdc1 (50 μm) together with Edc3 (150 μm; doped 1:100 with Edc3-OregonGreen). Scale bar, 50 μm.

Sequence alignments of the C-terminal region of the *S. pombe* Pdc1 protein with the C-terminal regions of the human, *D. melanogaster* and *A. thaliana* Edc4 proteins suggest the presence of a Ge-1_C_ domain^20^ in the Pdc1 protein, although the sequence identity (17 %) is very low (Figure S2 A). To confirm the presence of a Ge-1_C_ domain in Pdc1, we solved the crystal structure of residues 932 to 1076 to a resolution of 1.35 Å (PDB code: 4Q2S; Table S2). The structure displays a closely packed helical bundle, where the three N-terminal helices make an approximate 90-degree angle with the five C-terminal helices (Figure 5 A). The structure of the domain is very similar to the known structure of the Ge-1_C_ region of the *D. melanogaster* Ge-1 protein (Figure S2 B).

**Figure 5:**
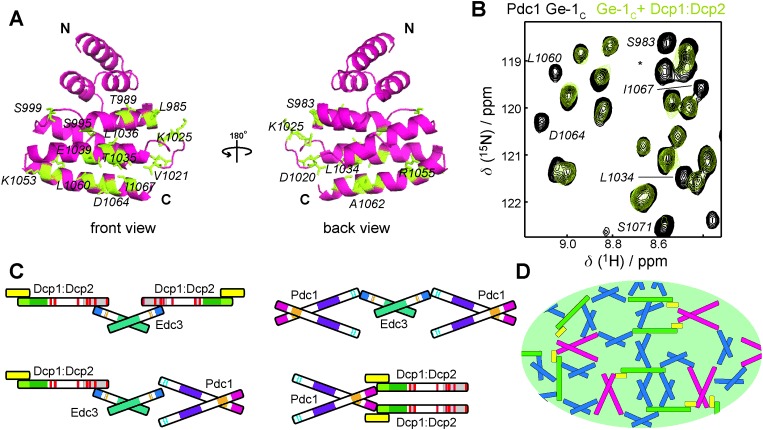
A) Ribbon diagram of the crystal structure of the Pdc1 Ge-1_C_ domain. Residues that interact with the decapping complex are highlighted in olive. B) NMR spectra of the Pdc1 Ge-1_C_ domain in the absence (black) and presence (olive) of the Dcp1:Dcp2 decapping complex that contains only the Dcp2 regulatory region. Resonances that experience chemical shift changes are labeled with the residue number. “*” refers to a resonance that is not assigned. C) The building blocks of the network of intermolecular interactions that leads to phase separation: One Edc3 dimer can interact with two decapping enzymes (left top), with two Pdc1 proteins (right top) or with one decapping complex and one Pdc1 dimer (bottom left). In addition one Pdc1 dimer can interact with two decapping complexes (bottom right). D) Schematic representation of how the building blocks displayed in (C) can be extended into an indefinite network of interactions. Edc3 blue, Dcp1 yellow, Dcp2 green, Pdc1 pink. These intermolecular interactions can result in phase separation (see Figures 2 and 4).

Previously, it was shown that the C-terminal 290 residues of Pdc1 interact with Dcp2.^10^ To probe if this interaction is direct, we performed NMR chemical shift titration experiments with the ^15^N labeled Pdc1 Ge-1_C_ domain and an unlabeled Dcp1:Dcp2 decapping complex (Dcp2 residues 1–95). We observe that resonance signals from residues that are located in the C-terminal helices of the Pdc1 Ge-1_C_ domain are significantly perturbed (Figure 5 B). This result indicates that Pdc1 exploits a surface at the far C-terminus of the protein to directly contact the Dcp1:Dcp2 decapping complex (Figure 5 A). Pdc1 can thus interact with Edc3 through a number of HLMs at its far N-terminal region and with the decapping complex through the Ge-1_c_ domain at its far C-terminal region. These results underscore the scaffolding function of Pdc1 and explain its importance for processing body formation. Addition of Pdc1 to Edc3 and Dcp2 (Figure 2 B) is thus expected to increase the strength of the interaction network, which is advantageous for the phase transitions process.

We have shown that it is possible to reproduce the in vivo phase separation behavior of the mRNA degradation machinery in a well-defined in vitro setting. Importantly, this bottom-up approach provides insights into the clustering behavior that could not have been obtained using genetic approaches owing to the highly redundant nature of the clustering process (Figure 5 C,D).

Additional mechanisms that promote the self-aggregation process of the mRNA degradation machinery will be exploited in the cell. These additional interactions will be able to lower the critical concentration required for phase separation to biologically relevant levels. In that regard, it is worth mentioning that it was recently shown that low complexity (LC) regions in the RNA binding protein FUS are able to induce phase transitions.^6^, ^12^, ^21^ Mechanistically, the LC regions aggregate through a mechanism that involves the formation of amyloid-like fibers, a feature that we did not detect in our current studies. This suggests that both processes that lead to phase separation are of a fundamentally different nature. Interestingly, short LC regions are also present in Dcp2 and Pdc1 and in other *S. pombe* processing body proteins, including LSm4, Pat1p, Sum2 (Scd6), Ste13 (Dhh1), and Exo2 (Xrn1).^22^ Future experiments will be able to shed light on how these LC regions, the HLM:Edc3 interactions and other specific intermolecular contacts (Figure 5 C) modulate the phase-transition process that underlies P-body formation (Figure 5 D). It has been suggested that biological systems might have evolved such that specific proteins are close to the phase separation conditions.^6^ Minor modifications in the valency and the affinity of the interacting partners by post-translational modifications or small changes in protein levels can then result in the sudden appearance or disappearance of cellular phase separation.^23^ Interestingly, the HLM rich region of Pdc1 contains a large number of phosphorylation sites^24^ that could potentially interfere with Edc3 binding and reduce the tightness of the intermolecular interaction network.

Our results form a starting point for future in vitro studies that address how the activity of the cellular enzymes can be influenced by local cellular molecular crowding. In addition, we anticipate that in vitro approaches similar to the one described herein can be exploited to address how other cellular granules are formed and how specific proteins are targeted to a specific class of granules.
