# Supplementary material for: In Vitro Reconstitution of a Cellular Phase-Transition Process that Involves the mRNA Decapping Machinery
Source: Angew Chem Int Ed Engl. 2014 May 26;53(28):7354–9. doi: 10.1002/anie.201402885 (PMC4320757; doi:10.1002/anie.201402885)
Supplement: Supplementary file 1 [file anie0053-7354-sd1.pdf]

Angewandte Chemie
Eine Zeitschrift der Gesellschaft Deutscher Chemiker

Supporting Information
© Wiley-VCH 2014
69451 Weinheim, Germany

In Vitro Reconstitution of a Cellular Phase-Transition Process that Involves the mRNA Decapping Machinery**

*Simon A. Fromm, Julia Kamenz, Erik R. Nöldeke, Ancilla Neu, Georg Zocher, and Remco Sprangers**

anie_201402885_sm_miscellaneous_information.pdf

Experimental section

Molecular Cloning and Protein Purification.

All plasmids used for protein overexpression in *E. coli* strain BL21 (DE3) Codon Plus RIL (Stratagene) were generated using standard cloning techniques (Supplementary Table S1A). Transformed cells were grown at 37 °C to an OD_{595} of 0.6-0.8 in LB and protein production was induced with 1 mM IPTG at 20 °C. Cells containing protein for NMR, ITC and X-ray experiments were lysed in buffer A (50 mM sodium phosphate, pH 7.5, 150 mM NaCl, 10 mM imidazole, 1 mM DTT) supplemented with 0.1 % Triton X-100, 0.5 mg/ml Lysozyme and 0.2 U/ml DNase I. The soluble fraction of the lysate was applied to Ni^{2+}-NTA resin equilibrated in buffer A. The retained protein fraction was eluted with buffer A containing 300 mM imidazole. After removal of the affinity tag with TEV protease, the protein was purified to homogeneity using size exclusion chromatography in SEC buffer (25 mM HEPES, pH 7.3, 125 mM NaCl, 1 mM DTT). HLM sequences were produced as MBP fusion constructs and were not digested with TEV protease.

NMR spectroscopy

Isotopically labeled proteins were grown in (H_2O or 70% D_2O based) M9 minimal medium containing $^{15}NH_4Cl$ as the sole nitrogen source and/ or ^{1}H ^{13}C glucose as the sole carbon source. NMR spectra were recorded at 30 °C on Bruker AVIII-600 or AVIII-800 spectrometers. Titration experiments were carried out with 0.1 mM ^{15}N labeled protein (Edc3 LSm or Pdc1 Ge-1_C) and 0.5 mM unlabeled protein (MBP-HLMs or Dcp1:$Dcp2_{regulatory}$; containing full length Dcp1 and residues 1-95 of Dcp2). The backbone resonances of the Pdc1 Ge-1_C domain were determined using HNCACB and HN(CO)CACB pulse-sequences. Spectra were processed with the NMRPipe/NMRDraw software suite [1], figures showing NMR spectra were prepared using NMRView (onemoonscientific.com) and figures showing molecular structures were made using Pymol (pymol.org).

ITC

ITC data were recorded at 30 °C with a TA Instruments NanoITC Low Volume calorimeter, where the cell contained the individual HLM sequences into which the Edc3 LSm domain was titrated. ITC data were fitted using in house written scripts using an independent binding model.

X-ray crystallography

Residues 923 to 1076 of the Pdc1 protein crystallized in 0.2 M ammonium acetate, 0.1 M HEPES pH 7.5, 25% (w/v) PEG 3350. Data collection at 100K was performed at beamlines X10SA and X06DA at the Swiss Light Source, Villigen, Switzerland. Three data sets were recorded to determine the structure of the Ge-1_C domain of the Pdc1 protein. A high-resolution data set resulted from data acquisition at 1.0 Å wavelength diffracting to 1.35 Å resolution. Two additional crystals of Pdc1 served for data recording at 1.7 Å and 2.07 Å wavelength to establish phases using the anomalous signal of proteinaceous sulfur. All data sets were reduced using the XDS/XSCALE package [2], are of spacegroup P21212 and contain one protein chain in the asymmetric unit. Due to non-isomorphism initial structure determination was performed using both long-wavelength data sets. The sulfur sites were determined by SHELXD [3] using the data set recorded at 2.07 Å. Phase refinement as implemented in SHARP/AUTOSHARP [4] was performed including the data sets recorded at 1.7 Å and 2.07 Å. The electron density map resulting from density modification was interpretable and allowed manual model building using COOT [5]. A crude model consisting of 75 residues was transferred to the high-resolution dataset using PHASER [6]. After initial simulated annealing, refinement proceeded in alternating rounds of manual model rebuilding in COOT and restrained coordinate and anisotropic B-factor refinement in REFMAC [7]. Solvent molecules were added with COOT:find_waters. The structure was validated using Molecular Graphics System, Version 1.5.0.4 Schrödinger, LLC. Ramachandran statistics are as follows: 96.9% in favored regions and 3.1% in additional allowed regions.

Phase separation and fluorescent microscopy.

Proteins for phase separation microscopy experiments were purified in a buffer that contained 25 mM phosphate, pH7.3, 200 mM NaCl and 1 mM DTT. Proteins were fluorescently labeled with Oregon Green in the same buffer, however, in the absence of DTT. To optimize the labeling efficiency Oregon Green maleimide (dissolved in dimethylformamide) was added in 5-10 fold excess. The coupling reaction was incubated for 2 h at room temperature and contained less than 1 % DMF v/v. The reaction was stopped by addition of DTT to a final concentration of 4 mM. Uncoupled dye was removed from the protein fraction using a desalting PD-10 column and the coupling efficiency was determined via UV/Vis spectroscopy. A C-terminal cysteine was added to proteins that contained no accessible natural cysteine. Phase separation experiments were carried out in special 96-well microscopy plates (MatTek Corp.). The final sample volume per well was 50-80 µl. Images were

recorded at room temperature on a Zeiss Axio Observer.Z1 coupled to an AxioCam MRM camera after mixing of the different components. All images were processed with ImageJ (NIH, USA).

***S. pombe* strains and imaging.**

S. pombe strains used in this study are listed in Supplementary Table S1B. PCR-based gene targeting was used to replace genes by gene fusions at their endogenous loci[8]. For overexpression of the HLM-1 the coding sequence of amino acids 242 to 291 of the *dcp2+* gene fused to the 13myc epitope[9] were cloned into a pDual vector[10] under the control of the constitutive *adh1* promoter[11] and integrated into the *leu1* locus. Cell growth and imaging conditions for the visualization of P-bodies have been described previously[12].

SUPPORTING FIGURES

Figure S1

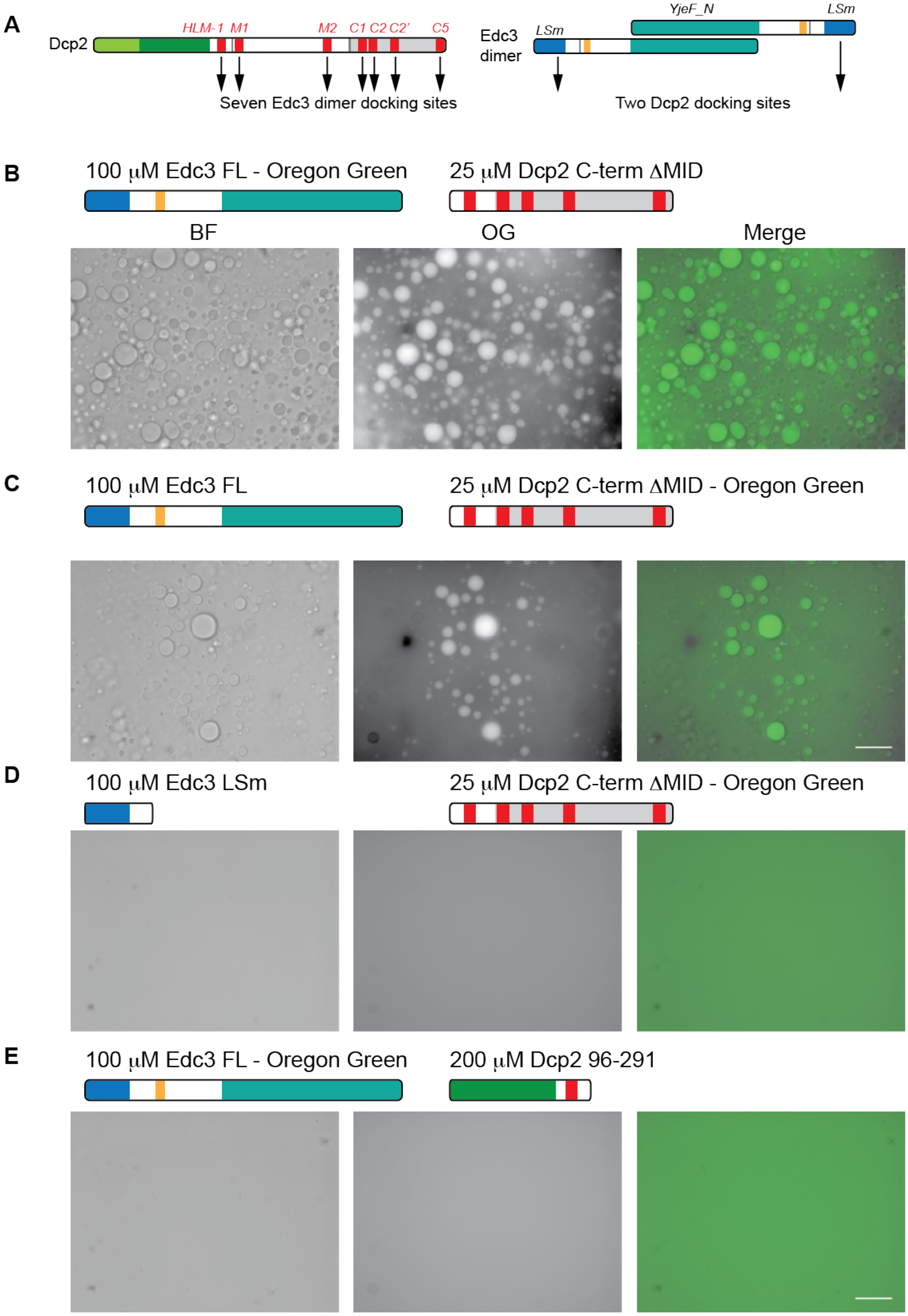

Figure S1 Phase separation of the Dcp2 and Edc3 proteins. (A): The Dcp2 protein has seven docking sites (HLM sequences) for the LSm domain of Edc3. The dimeric Edc3 protein has two LSm domains that can interact with HLM sequences. (B, C): Both the Edc3 and the Dcp2 protein are enriched in the droplet phase. In (B), the Edc3 protein is fluorescently labeled with Oregon green, in (C) the Dcp2 protein is fluorescently labeled with Oregon green. (D): A monomeric version of the Edc3 protein is not able to undergo phase transitions with Dcp2. (E) A Dcp2 protein that contains a single HLM is not able to undergo phase transitions with the dimeric Edc3 protein. The proteins that are fluorescently labeled are indicated. The scale bars correspond to 50 µm.

Figure S2

A

```
S. pombe (Pdc1)          932  AQGIAESLRRLKEYVKAGSVKECV--AEWCNMPSVAGFDVLSEISYDRMLENC---SNLLLLTFIYHISLLDSVDDDR
D. melanogaster (Ge-1)  1219  G-------DSIKQLLMAGQINKAFHQALLANDLGLVEFTLRHTDSNQAFAPEGCRLEQKVLLSLIQQISA-DMTN--H
H. sapiens (Edc4)       1051  HLDCQAQQAHILQLLQQGHLNQAFQQALTAADLNLVLYVCETVDPAQVFGQPPCPLSQPVLLSLIQQLAS-DLGT--R
A. thaliana (VSC)       1169  VEAPMDPTTELSRLISERKYEESFTSALQRSDVSIVSWLCSQVDLRGLLAMNPLPLSQGVLLSLLQQLAC-DISK--D

S. pombe (Pdc1)               LSKRMEYISRICLNIDVNDPKVETVVHPVLTLTREAL---LRQSEFFSPIFK-RRLVVLLRALDGKISEISVASSN  1076
D. melanogaster (Ge-1)        NELKQRYLNEALLAINMADPITREHAPKVLTELYRNCQQFIKNSPKNSQFSNVR---LLMKAIITYRDQLK------  1354
H. sapiens (Edc4)             TDLKLSYLEEAVMHLDHSDPITRDHMGSVMAQVRQKLFQFLQAEPHNSLGKAARRLSLMLHGLVTPSLP-------  1194
A. thaliana (VSC)             TSRKLAWMTDVVAAINPSDQMIAVHARPIFEQVYQILHHH-RNAP-GSDVSAIR---LIMHVINSMLMGCK------  1309
```

B

S. pombe Pdc1 Ge-1$_C$

D. melanogaster Ge-1 Ge-1$_C$ (PDB: 2VXG)

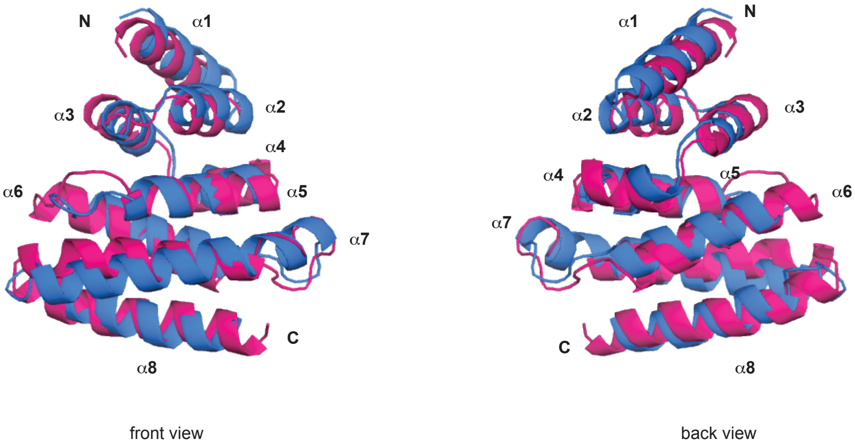

Figure S2 (A): Sequence alignment of the *S. pombe*, *D. melanogaster*, *H. sapiens* and *A. thaliana* Edc4 homologs. The residue numbers are indicated and the alignment is colored according to residue type, where the color intensity correlates with sequence conservation. (B): Overlay of the structures of the Pdc1 Ge-1$_C$ domain and the *D. melanogaster* Ge-1$_C$ domain of the Ge-1 protein [13]. The view is identical to the view in Figure 5 of the main paper.

SUPPORTING TABLES

**Protein/Protein complex**	**Residues**	**Purification/Solubility-Tag**	**Used in Figure**	**Internal reference**
Edc3	1-94	N-His$_6$-TEV	1B, 1C, 1D, 4B, 4C, 4D, S1D	194
Dcp2	242-291 (HLM-1)	N-His$_6$-MBP-TEV	1B, 1C, 1D, 2E	226
Dcp2	292-318 (HLM-M1)	N-His$_6$-MBP-TEV	1B, 1D	882
Dcp2	482-504 (HLM-M2)	N-His$_6$-MBP-TEV	1B, 1D	895
Dcp2	553-576 (HLM-C1)	N-His$_6$-MBP-TEV	1B, 1C, 1D	302
Dcp2	577-604 (HLM-C2)	N-His$_6$-MBP-TEV	1B, 1C, 1D	880
Dcp2	605-640 (HLM-C2')	N-His$_6$-MBP-TEV	1B, 1D	881
Dcp2	709-741 (HLM-C5)	N-His$_6$-MBP-TEV	1B, 1D	329
Edc3	1-454	N-His$_6$-TEV	2A-E, 4E, S1B, S1C, S1E	180
Dcp2	553-741	N-His$_6$-TEV	2A-C	68
Dcp2<br>Dcp2 C-term ΔMid	242-741Δ290-552	N-His$_6$-TEV	2B, 2E, S1B, S1D	752
Dcp2 C742<br>Dcp2 C-term ΔMid	242-741Δ290-552 (cysteine at C-terminus)	N-His$_6$-TEV	S1C	764
Dcp1:Dcp2 C742<br>Dcp1:Dcp2 ΔMid	1-127 (Dcp1), 1-741Δ290-552 (cysteine at C-terminus)	N-His$_6$-TEV	2D	769
Control	MBP	N-His$_6$-MBP-TEV	1B, 4B	189
Pdc1	1-1076	N-His$_6$-TEV	text	829
Pdc1-GST-Pdc1	1-105 (Pdc1)-GST-880-1076 (Pdc1)	N-His$_6$-TEV	4E	928
Pdc1	932-1076	N-His$_6$-TEV	5A, 5B, 5C	892
Dcp1:Dcp2	1-127 (Dcp1), 1-95 (Dcp2)	N-His$_6$-TEV (Dcp1), none (Dcp2)	5B, 5C	50
Dcp2 C292	96-291 (cysteine at C-terminus)	N-His$_6$-TEV	S1E	767
Pdc1	1-25 (HLM-N1)	N-His$_6$-MBP-TEV	4B, 4D	884
Pdc1	49-73 (HLM-N2)	N-His$_6$-MBP-TEV	4B, 4C, 4D	885
Pdc1	80-104 (HLM-N3)	N-His$_6$-MBP-TEV	4B, 4D	886

Table S1A: List of proteins used in this study, including construct boundaries and affinity/ solubility tags.

Figure 3A		
RS001	*h+*	*leu1 ade6-M216 dcp2+-GFP<<kanR edc3+-mCherry<<natR*
RS035	*h+*	*leu1 ade6-M216 dcp2+-GFP<<kanR edc3+-mCherry<<natR leu1+<<Padh1-dcp2-242-292-13myc*

Figure 3B		
RS037	*h+*	*leu1 ade6-M210 lsm7+-GFP<<kanR edc3+-mCherry<<natR*
RS039	*h+*	*leu1 ade6-M210 lsm7+-GFP<<kanR edc3+-mCherry<<natR leu1+<<Padh1-dcp2-242-292-13myc*

Table S1B: List of *S. pombe* strains used in this study.

	Pdc1 Ge-1_C domain
**Data collection**	
Space group	P 21 21 2
Cell dimensions	
a, *b*, *c* (Å)	47.33 77.84 36.51
a, b, g (°)	90 90 90
Resolution (Å)	40-1.35
R_{meas}	5.9(102.5)
R_{merge}	5.4(94.2)
I / sI	17.19(2.03)
Completeness (%)	99.7(100.0)
Redundancy	6.3(6.4)
**Refinement**	
Resolution (Å)	1.35
No. reflections	30287
R_{work} / R_{free}	18.5/21.2
No. atoms	
Protein	1092
Water	70
B-factors	
Protein	20.1
Water	29.9
R.m.s. deviations	
Bond lengths (Å)	0.01
Bond angles (°)	1.43

Values in parentheses are for highest-resolution shell.

Table S2: X-ray data collection and refinement statistics.

SUPPORTING REFERENCES

[1] F. Delaglio, S. Grzesiek, G. W. Vuister, G. Zhu, J. Pfeifer, A. Bax, *Journal of biomolecular NMR* **1995**, *6*, 277-293.

[2] W. Kabsch, *Acta crystallographica. Section D, Biological crystallography* **2010**, *66*, 125-132.

[3] G. M. Sheldrick, *Acta crystallographica. Section D, Biological crystallography* **2010**, *66*, 479-485.

[4] G. Bricogne, C. Vonrhein, C. Flensburg, M. Schiltz, W. Paciorek, *Acta crystallographica. Section D, Biological crystallography* **2003**, *59*, 2023-2030.

[5] P. Emsley, B. Lohkamp, W. G. Scott, K. Cowtan, *Acta crystallographica. Section D, Biological crystallography* **2010**, *66*, 486-501.

[6] A. J. Mccoy, R. W. Grosse-Kunstleve, P. D. Adams, M. D. Winn, L. C. Storoni, R. J. Read, *J Appl Crystallogr* **2007**, *40*, 658-674.

[7] G. N. Murshudov, P. Skubak, A. A. Lebedev, N. S. Pannu, R. A. Steiner, R. A. Nicholls, M. D. Winn, F. Long, A. A. Vagin, *Acta crystallographica. Section D, Biological crystallography* **2011**, *67*, 355-367.

[8] J. Bahler, J. Q. Wu, M. S. Longtine, N. G. Shah, A. McKenzie, 3rd, A. B. Steever, A. Wach, P. Philippsen, J. R. Pringle, *Yeast* **1998**, *14*, 943-951.

[9] M. S. Longtine, A. McKenzie, 3rd, D. J. Demarini, N. G. Shah, A. Wach, A. Brachat, P. Philippsen, J. R. Pringle, *Yeast* **1998**, *14*, 953-961.

[10] A. Matsuyama, A. Shirai, Y. Yashiroda, A. Kamata, S. Horinouchi, M. Yoshida, *Yeast* **2004**, *21*, 1289-1305.

[11] M. McLeod, M. Stein, D. Beach, *The EMBO journal* **1987**, *6*, 729-736.

[12] S. A. Fromm, V. Truffault, J. Kamenz, J. E. Braun, N. A. Hoffmann, E. Izaurralde, R. Sprangers, *The EMBO journal* **2012**, *31*, 279-290.

[13] M. Jinek, A. Eulalio, A. Lingel, S. Helms, E. Conti, E. Izaurralde, *Rna* **2008**, *14*, 1991-1998.
